# Evaluating ^18^F-FDG PET-CT for Regional Lymph Node Assessment in Advanced Upper Tract Urothelial Carcinoma

**DOI:** 10.1016/j.euros.2026.04.013

**Published:** 2026-05-05

**Authors:** Ragnheidur Fridriksdottir, Ioannis Patras, Axel Gerdtsson, Kevin Sandeman, Petter Kollberg, Fredrik Liedberg, Johannes Bobjer, Elin Trägårdh

**Affiliations:** a Department of Clinical Physiology and Nuclear Medicine, Skåne University Hospital, Lund/Malmö, Sweden; b Department of Translational Medicine, Lund University, Malmö, Sweden; c Department of Urology, Skåne University Hospital, Malmö, Sweden; d Department of Pathology, Medical Services, Skåne University Hospital, Malmö, Sweden

**Keywords:** CT, Diagnostic accuracy, ^18^F-FDG PET-CT, Lymph node metastasis, Preoperative staging, Upper urinary tract urothelial carcinoma (UTUC), Template-based regional lymphadenectomy

## Abstract

**Background:**

Preoperative lymph node staging in upper tract urothelial carcinoma (UTUC) using ^18^F-Fluorodeoxyglucose (FDG) positron emission tomography combined with computed tomography (PET-CT) is sparsely reported.

**Objective:**

This study investigates the diagnostic accuracy of PET-CT and conventional computed tomography (CT) for detecting regional lymph node metastases in patients with UTUC, using histopathology as the reference standard.

**Design setting and participants:**

A total of 75 consecutive patients with UTUC, who underwent radical nephroureterectomy (RNU) with regional lymphadenectomy (rLAE) between 2014 and 2024, were retrospectively analyzed. All patients underwent preoperative PET-CT.

**Outcome measurements and statistical analysis:**

Accuracy for PET-CT and CT was compared with histopathology in fractionated lymphadenectomy specimens in the total cohort and in the subgroup that did not receive preoperative chemotherapy (PC).

**Results and limitations:**

Of the 75 patients, 48 were male (64%), 23 (31%) received PC, and 18 (24%) had histologically confirmed lymph node metastases. For all patients, results for PET-CT and their 95% confidence intervals were as follows: sensitivity 83% (59–96), specificity 70% (57–82), and positive likelihood ratio (LR+) 2.8 (1.8–4.4), compared with 39% (17–64), 83% (70–91), and 2.2 (1.0–5.0) for CT, respectively. In the subgroup of 52 patients who did not receive PC, PET-CT showed a sensitivity of 75% (43–95), specificity of 90% (76–97), and LR+ of 7.5 (2.8–20.1), while CT yielded 25% (6–57), 93% (80–98), and 3.3 (0.8–14.4), respectively. The primary limitation of the study is the limited sample size, particularly the small number of patients with confirmed nodal metastases.

**Conclusions:**

Preoperative PET-CT demonstrates promising diagnostic accuracy for regional lymph node staging in patients with UTUC.

## Take Home Message

Preoperative positron emission tomography combined with computed tomography shows promising accuracy for regional nodal staging in patients with upper tract urothelial cancer.

ADVANCING PRACTICE **What does this study add?**

The study supports the use of positron emission tomography combined with computed tomography for preoperative lymph node staging.

**Clinical Relevance**

^8^F-FDG PET/CT improves preoperative nodal staging in UTUC compared to conventional CT, with substantially higher sensitivity (83% vs 39%), enabling better identification of patients with occult nodal disease. More accurate detection of nodal metastases may directly impact treatment selection, particularly in guiding the use of systemic induction therapy in an era of increasingly effective perioperative treatments. However, given the small sample size and heterogeneous cohort, these findings should be interpreted cautiously and require prospective validation before routine clinical adoption. Associate Editor: Maria Carmen Mir.

**Patient Summary**

PET-CT is a promising tool that can help doctors better detect whether cancer has spread to nearby lymph nodes before surgery for UTUC.

## 1 Introduction

Upper tract urothelial carcinoma (UTUC) manifests as an advanced disease (clinical stage ≥ T2) in approximately 45% of all patients [1]. Primary lymph node metastases are observed in 14–40% of these patients with advanced disease and are associated with worse overall survival [2]. For patients with high-risk UTUC, guidelines recommend radical nephroureterectomy (RNU) with template-based regional lymphadenectomy (rLAE) [3], [4]. Although computed tomography (CT) urography is commonly used for staging, its sensitivity for detecting lymph node involvement in UTUC is low [5]. The diagnostic performance of ^18^F-Fluordeoxyglucose (FDG) positron emission tomography combined with computed tomography (PET-CT) for regional lymph node metastases in patients with UTUC, with histopathology as reference, has been investigated in only a few studies (*n* = 120 patients) [6], [7], [8], with reported sensitivity up to 82% [7]. In the wake of level 3 evidence supporting adjuvant chemotherapy after RNU [9], hypothesis-generating findings suggest that node-positive patients, in particular, benefit from such therapy [10], further underscoring the importance of accurate nodal staging.

In this study, data from a prospective multicenter lymph node mapping trial were used to retrospectively assess the diagnostic accuracy of preoperative PET-CT and CT compared to histopathology in fractionated lymphadenectomy specimens in patients with invasive UTUC in the renal pelvis or proximal ureter who were subjected to rLAE.

## 2 Patients and methods

### 2.1 Study population

A total of 75 consecutive patients with UTUC who underwent preoperative PET-CT between December 8, 2014 and February 20, 2024 were retrospectively included from an ongoing, prospective study evaluating template-based rLAE (ISRCTN83155790). Of them, 71 patients were diagnosed with clinical tumor stage ≥cT2. The remaining 4 patients had <cT2; however, they showed clinical evidence of lymph node metastases on preoperative imaging. For patients with clinical evidence of lymph node metastases who were eligible for platinum-based chemotherapy, the primary PET-CT and CT, which were performed before start of systemic induction treatment, were used. Patient characteristics and clinicopathological data, including classification according to tumor node metastases 2017 [11], were retrieved by chart review.

### 2.2 PET-CT imaging and analysis

PET-CT was performed at four Swedish hospitals using Philips Gemini TF (Philips Medical systems, Cleveland, OH) and GE Discovery (690, 710, MI) (GE Healthcare, Milwaukee, WI, USA).

Patients were prepared for imaging by fasting for 4 h. A glucose level below 10 mmol/l was ensured before administering injection with 4 MBq/kg of FDG. Of the 75 patients, 50 (67%) were scanned 60 min after injection and the remaining 25 patients were scanned 120–140 min after administration of FDG, with 20 mg furosemide administered intravenously 60 min after FDG administration. The CT used for attenuation correction and anatomic correlation was of low dose in 46 patients (61%) and of diagnostic quality with or without intravenous contrast in 29 patients (39%). For patients whose PET-CT included low-dose CT, most had undergone a diagnostic-quality CT urography within 6 wk prior to PET-CT; however, 12 patients had only low-dose CT available for assessment of short axis lymph node diameter (SALND).

All PET-CT and CT investigations were retrospectively assessed for regional lymph node metastases in a blinded manner by one physician experienced in nuclear medicine (R.F.), with only information about tumor laterality available.

PET-CT was considered positive if at least one regional hypermetabolic lymph node was present. CT was considered positive if a regional lymph node with a SALND of ≥10 mm was present [12], [13], [14]. The same criteria were applied for positivity when analyzing per anatomical area defined by the fractions of the template used for lymphadenectomy [14]. For lymph node metastases located on the border between two template fractions, a consensus reading (R.F. and I.P.) was performed using information from the operative report (applied for all image analyses).

### 2.3 Preoperative chemotherapy

Systemic treatment recommendations changed during the study. Early in the series, neoadjuvant platinum-based chemotherapy was considered for cisplatin-eligible patients with ≥cT2 disease, given the low eligibility after RNU [15]. Induction chemotherapy for node-positive disease became standard later [16], and was applied consistently across hospitals.

### 2.4 Surgery and pathology

All patients underwent side-specific template-based rLAE [17], 65 (87%) at Skåne University Hospital, nine (12%) at Helsingborg County Hospital, and one (1%) at Umeå University Hospital. Templates included four fractions for right-sided and two for left-sided tumors, along the infrarenal aorta and vena cava inferior, defined by anatomical landmarks as described by Bobjer et al [17]. Additional areas were dissected at the discretion of the urologist when metastases were suspected outside of the templates, either at PET-CT or intraoperatively. The lymph node yield and number of metastatic lymph nodes per fraction in the histopathologic specimens were registered. The largest diameter of metastases within the fractionated specimen was reassessed by a uropathologist (K.S).

### 2.5 Statistical analysis

Continuous variables were expressed as medians with interquartile ranges (IQRs) and proportions as percentages. The diagnostic accuracy of PET-CT and CT, with histopathology as the reference method, was calculated as sensitivity, specificity, positive and negative predictive values (PPV and NPV), and positive and negative likelihood ratios (LR+ and LR−) for each patient and per anatomical fraction with an identifiable lymph node on imaging, and separately for the subgroup who did not receive preoperative chemotherapy (PC), since a complete response after PC may affect the accuracy of preoperative staging. The 95% confidence intervals (CIs) were calculated using the Clopper-Pearson method. Fisher’s exact test was used to compare differences in sensitivity and specificity of PET-CT and CT in regards to: prior invasive investigation instrumentation within 6 wk before PET-CT (*n* = 13) compared to those without such procedures (*n* = 62) for both PET-CT and CT; accumulation time of 120 min (*n* = 25) compared to 60 min (*n* = 50) for PET-CT; and low-dose CT without intravenous contrast for SALND assessment (*n* = 12) compared to full-dose CT (*n* = 63). Analyses were performed in STATA SE 18.0.

The study was approved by the Research Ethics Board at Lund University, Malmö, Sweden (Dnr 2013/321; 2016/645; 2018/84). All patients signed an informed consent.

## 3 Results

### 3.1 Patient characteristics

Patient characteristics, tumor, and treatment details are summarized in Table 1 for all 75 patients and for the subgroup who did not receive PC (*n* = 52). Metastases were detected in the lymphadenectomy specimens in 18 (24%) of the total cohort of 75 patients and in 12 (23%) of the subgroup of 52 patients who did not receive PC. Two of the patients without UTUC lymph node metastases had other malignancies in the lymph nodes on histopathology: one had nodal marginal zone lymphoma and the other had chronic lymphocytic leukemia. The median number of excised lymph nodes per patient was 12 (IQR 7–21), with additional descriptive information in Table 2. Thirteen patients (17%) had lymph nodes resected outside the predefined unilateral template, with metastasis found in one patient. Histopathology results from RNU specimens showed urothelial carcinoma for 70 patients, including 1 patient with squamous cell differentiation and one with nested histological subtype. The remaining 5 patients among those who received PC had no remaining tumor in the specimen (ypT0).

**Table 1:** Patient, tumor, surgical approach, and treatment characteristics of the study population for all patients (*n* = 75) and for the subgroup who did not receive preoperative chemotherapy (*n* = 52)

	All patients (%)patients (%)	rLAE without PC (%)(%))%) (%) (%)patients without PC PC (n=52)
Median age (IQR) yr	72 (65–76)	73 (66–77)
Gender		
Male	48 (64)	31 (60)
ASA score		
1	17 (23)	11 (21)
2	38 (51)	28 (54)
3	20 (27)	13 (25)
Tumor side		
Right side	31 (41)	23 (44)
Left side	44 (59)	29 (56)
Tumor location		
Renal pelvis	59 (79)	40 (77)
Proximal ureter	10 (13)	8 (15)
Multifocal	6 (8.0)	4 (7.7)
Clinical T stage		
cCIS	1 (1.3)	0 (0)
cT1	3 (4.0)	1 (1.9)
cT2	17 (23)	12 (23)
cT3	51 (68)	37 (71)
cT4	4 (5.3)	2 (3.9)
Urine cytology Paris classification a		
2	21 (28)	14 (27)
3	34 (45)	26 (50)
4 or 5	20 (27)	12 (23)
Type of PC		
Neoadjuvant ddMVAC	3 (13)	
Induction ddMVAC	10 (44)	
Induction GCa	10 (44)	
Invasive instrumentation at staging (within 6 wk prior to imaging) b		
Ureteroscopy ± selective cytology	11 (14)	8 (15)
Nephrostomy	2 (2.7)	2 (3.9)
Double-J catheter	1 (1.3)	1 (1.9)
Median (IQR) d between imaging and rLAE	60 (39–102)	48 (32–62)
Surgical approach of RNU and rLAE		
Open RNU and rLAE	52 (69)	36 (67)
Robot-assisted laparoscopic RNU and rLAE	17 (23)	14 (29)
Other c	6 (8.0)	2 (3.9)

ASA = American Society of Anesthesiologists; cCIS = carcinoma in situ; ddMVAC = methotrexate, vinblastine, doxorubicin, and cisplatin; GCa = gemcitabine and carboplatin; IQR = interquartile range; PC = preoperative chemotherapy; rLAE = regional lymphadenectomy; RNU = radical nephroureterectomy; UTUC = upper tract urothelial carcinoma.

a Voided or obtained selectively from the upper urinary tract.

b In total 13 patients; however, 1 patient was subjected to both ureteroscopy and nephrostomy within 6 wk prior to PET-CT.

c Other surgical approaches: 2 patients operated with consolidating rLAE after receiving PC after RNU; 2 patients operated with concomitant cystectomy as well as RNU and rLAE (one of whom had received PC); 1 patient operated with consolidating robot-assisted laparoscopic lymphadenectomy after PC; 1 patient with bilateral UTUC was operated with open right-sided RNU and bilateral rLAE along with left-sided ureterectomy and an autotransplantation of left kidney (only the right-sided UTUC was considered in the study cohort). Imaging denotes ^18^F-FDG PET-CT (^18^F-fluorodeoxyglucose–positron emission tomography with computed tomography).

**Table 2:** Description of lymph nodes and metastases in the regional lymphadenectomy specimen

	All patients	rLAE without PC
Median number of lymph nodes excised (IQR)	12 (7–21)	15 (7–24)
Median number of metastatic lymph nodes (IQR)	2 (1–3)	2 (1–3)
Median diameter of largest lymph node metastasis (IQR) mm	9.5 (5–15)	9.5 (5.5–14)

IQR = interquartile range; mm = millimeter; PC = preoperative chemotherapy; rLAE = regional lymphadenectomy.

### 3.2 Diagnostic performance

In the total cohort, PET-CT was positive in 32 patients (43%), with 15 (47%) classified as true positives (TPs). CT was positive in 17 patients (23%), with 7 (41%) classified as TPs. All TPs on CT were positive on PET-CT. Among the 18 patients with histopathologically confirmed metastases, PET-CT was false negative (FN) in three patients (16%), while CT was FN in 11 patients (61%). One patient who was classified as false positive (FP) on CT and true negative (TN) on PET-CT showed nodal marginal zone lymphoma on histopathology, and one patient who had chronic lymphocytic leukemia was classified as TN on both PET-CT and CT. Out of the 23 patients who received PC, 13 were positive on PET-CT, of whom six had histologically confirmed nodal metastases. For the entire cohort, results of the diagnostic accuracy and their 95% CI for PET-CT demonstrated a sensitivity of 83% (59–96), specificity of 70% (57–82), and LR+ of 2.8 (1.8–4.4). For CT, the corresponding values were 39% (17–64), 83% (70–91), and 2.2 (1.0–5.0), respectively.

In the non-PC subgroup (*n* = 52), PET-CT was positive in 13 patients (25%), with nine (69%) classified as TP. CT was positive in six patients (12%), with three (50%) classified as TP. Among the 12 patients with confirmed lymph node metastases, PET-CT was FN in three cases (25%), while CT was FN in 9 (75%) cases. Results on diagnostic accuracy with 95% CI for PET-CT were as follows; sensitivity 75% (43–95), specificity 90% (76–97), and LR+ 7.5 (2.8–20.1), compared to 25% (6–57), 93% (80–98), and 3.3 (0.8–14.4) for CT, respectively.

All results on diagnostic accuracy, including PPV, NPV, and LR− for both total cohort and non-PC subgroup are displayed in Table 3.

**Table 3:** Diagnostic accuracy analysis per patient in all patients (*n* = 75) and the subgroup who underwent surgery without PC (*n* = 52)

	PET-CT		CT-scan	
	All patients	rLAE without PC	All patients	rLAE without PC
True positive	15	9	7	3
False negative	3	3	11	9
True negative	40	36	47	37
False positive	17	4	10 a	3
Diagnostic accuracy (CI)				
Sensitivity	83 (59–96)	75 (43–95)	39 (17–64)	25 (6–57)
Specificity	70 (57–82)	90 (76–97)	83 (70–91)	93 (80–98)
PPV	47(29–65)	69 (39–91)	41 (18–67)	50 (12–88)
NPV	93 (81–99)	92 (79–98)	81 (69–90)	80 (66–91)
LR+	2.8 (1.8–4.4)	7.5 (2.8–20.1)	2.2 (1.0–5.0)	3.3 (0.8–14.4)
LR−	0.2 (0.1–0.7)	0.3 (0.1–0.7)	0.7 (0.5–1.1)	0.8 (0.6–1.1)

CI = confidence interval; CT = computed tomography; FP = false positive; LR+ = positive likelihood ratio; LR− = negative likelihood ratio; NPV = negative predictive value; PC = preoperative chemotherapy; PET-CT = positron emission tomography combined with computed tomography; PPV = positive predictive value; rLAE = regional lymphadenectomy.

PET-CT was subjectively interpreted, and CT was considered positive when short-axis lymph node diameter was ≥10 mm.

a One FP case on CT was due to nodal enlargement from nodal marginal zone lymphoma.

No statistically detectable differences in sensitivity and specificity for PET-CT or CT were observed between patients who underwent invasive upper tract instrumentation prior to imaging, which may be due to heterogeneity in imaging protocols, differences in accumulation time (for PET-CT), and variations in quality of CT (data not shown); however, these analyses were likely underpowered, and meaningful differences cannot be excluded.

Diagnostic accuracy calculated per anatomical fraction with identifiable lymph nodes on imaging was similar to the per-patient analysis, both in the full cohort and in the non-PC subgroup. These results are presented in Supplementary Table 1.

## 4 Discussion

The results of this multicenter study of 75 consecutive patients with UTUC demonstrate that preoperative PET-CT is a useful lymph node staging tool with promising diagnostic accuracy.

Our findings in the non-PC subgroup align with previous reports, such as Voskuilen et al, showing similar diagnostic performance of PET-CT in 62 patients undergoing preoperative PET-CT and subsequent rLAE without PC [7]. However, this study adds methodological rigor through blinded imaging review, standardized template-based surgeries as recommended by guidelines [3], and a higher median lymph node yield (12 vs 6). The use of these templates has prospectively been shown to capture majority of lymph node metastases [2], [3], [17]. To our knowledge, only two additional studies have reported PET-CT accuracy for lymph node staging using histopathology from rLAE as reference [6], [8]. The lower sensitivity estimates in those studies (60% and 40%, respectively) are difficult to interpret given the low number of patients (*n* = 5 [6]) and the low number of nodes with histologically verified lymph node metastases (*n* = 5 [8]), as well as lack of reporting the number of lymph node-positive patients, respectively.

Low sensitivity and high specificity on CT in the non-PC subgroup in this study are comparable to the results on accuracy of conventional CT for lymph node staging assessed by Pallauf et al in a large retrospective multicenter study including 865 patients with UTUC who underwent surgery without PC, with histopathology of the lymphadenectomy specimens as reference [5]. A single FP case on CT in our cohort was due to nodal enlargement from nodal marginal zone lymphoma, illustrating the limitation of size-based CT criteria in the presence of alternative nodal pathology, which minimally affects the diagnostic accuracy of CT.

From a clinical perspective, accurate nodal staging has significant implications. The higher sensitivity, similar specificity, and improved LR+ for PET-CT compared to those of CT allow detection of additional patients with metastatic disease preoperatively, enabling tailored treatment strategies, including systemic induction therapy [16], which are increasingly relevant in the era of novel systemic therapies. An altered treatment plan based on PET-CT findings was observed in one out of five patients in the study by Tanaka et al [8], which is in the same range as the 17% FN patients on CT in this series. Altered treatment based on PET-CT findings has been reported for bladder cancer prior to radical cystectomy in 18–68% of patients [18], where publication of the PET MUSE trial (Impact of Positron Emission Tomography (PET) imaging in Muscle-invasive Urothelial carcinoma of the Bladder Staging, NCT02462239) exploring such endpoints in a randomized bladder cancer trial is eagerly awaited. In that trial, and in this clinical practice applying PET-CT in patients with UTUC, altered treatment is mainly based on additional findings compared to conventional CT, prioritizing high specificity and LR+ over high NPV.

CT is more widely available and less costly compared to PET-CT, but for each patient the correct identification of a metastatic disease and subsequent initiation of PC offer an individualized and tailored treatment approach. This is increasingly relevant when entering an era with a new and more efficient first line therapy with enfortumab vedotin plus pembrolizumab [19]. Although not investigated in this study, use of PET-CT to improve detection of non-regional lymph node metastases and synchronous distant oligometastases is also relevant when the curative window expands with the use of new systemic therapies [20]. Response evaluation with PET-CT after induction chemotherapy has been shown to predict survival in muscle-invasive bladder cancer [21], but to our knowledge it has not been assessed in UTUC. The clinical assessment of response on systemic induction therapy with PET-CT or CT is beyond the scope of this study, more available systemic treatment lines potentially further increase the utility of PET-CT to investigate response.

Heterogeneity in PET-CT acquisition protocols may have introduced measurement variability and influenced diagnostic accuracy estimates. Given the small cohort and low number of patients with confirmed nodal metastases (*n* = 18), the study is underpowered to detect clinically meaningful differences between these heterogenous subgroups, and absence of significant differences should be interpreted cautiously. The blinded review by a single reader made it impossible to address the issue with interobserver variation when interpreting PET-CT examinations.

An important methodological consideration is that patients receiving PC may experience complete nodal response between imaging and surgery, resulting in misclassification of PET-CT findings as FP. This leads to an underestimation of specificity, PPV, and LR+ in the full cohort, and the magnitude of this effect cannot be determined. In the group that received PC, 13 patients were positive on PET-CT and six of those had metastases on histopathology. The remaining 7 (54%) patients may reflect chemotherapy-related nodal response; however, baseline FPs cannot be excluded.

Although restricting the analysis to the non-PC subgroup avoids this endpoint-related misclassification and provides a more internally valid estimate of baseline staging performance, it likely selects a subgroup with lower metastatic burden. This limits generalizability because PET-CT in real-world setting is used to identify patients suitable for PC (induction therapy in node-positive disease) [16]. Therefore, the non-PC subgroup offers a cleaner estimate of diagnostic performance, whereas the full cohort better reflects the population in which PET-CT is clinically applied; both aspects should be acknowledged when interpreting the results.

The diagnostic accuracy of PET-CT compared to that of CT would ideally be studied in a prospective setting, but given the rarity of locally advanced UTUC, conducting randomized controlled trials investigating, for example, altered treatment as primary outcome, is challenging. Nevertheless, our findings align with prior studies [7], [8] supporting the value of PET-CT for nodal staging in patients with UTUC. In addition, PET-CT has shown superiority in detecting distant metastases in muscle-invasive bladder cancer [22], which likely also can be generalized to the setting of locally advanced UTUC.

## 5 Conclusions

Preoperative PET-CT demonstrates promising diagnostic accuracy for regional nodal staging in patients with UTUC. Though these findings are consistent with previously published observations, cautious interpretation is warranted given the descriptive nature of the analyses and the limited number of patients with nodal metastases. Further prospective studies are needed to validate the potential role of PET-CT in guiding systemic treatment decisions.

  ***Author contributions:*** Ragnheidur Fridriksdottir had full access to all the data in the study and takes responsibility for the integrity of the data and the accuracy of the data analysis.

  *Study concept and design*: Fridriksdottir, Patras, Gerdtsson, Liedberg, Bobjer, Trägårdh.

*Acquisition of data*: Fridriksdottir, Patras, Sandeman, Kollberg, Gerdtsson, Liedberg, Bobjer, Trägårdh.

*Analysis and interpretation of data*: Fridriksdottir, Patras, Sandeman, Kollberg, Gerdtsson, Liedberg, Bobjer, Trägårdh.

*Drafting of the manuscript*: Fridriksdottir, Patras, Sandeman, Kollberg, Gerdtsson, Liedberg, Bobjer, Trägårdh.

*Critical revision of the manuscript for important intellectual content*: Fridriksdottir, Patras, Sandeman, Kollberg, Gerdtsson, Liedberg, Bobjer, Trägårdh.

*Statistical analysis*: Fridriksdottir.

*Obtaining funding*: Trägårdh, Liedberg, Gerdtsson, Bobjer.

*Administrative, technical, or material support*: Fridriksdottir, Patras, Sandeman, Kollberg, Gerdtsson, Liedberg, Bobjer, Trägårdh.

*Supervision*: Gerdtsson, Liedberg, Bobjer, Trägårdh.

*Other* (specify): None.

  ***Financial disclosures:*** Ragnheidur Fridriksdottir certifies that all conflicts of interest, including specific financial interests and relationships and affiliations relevant to the subject matter or materials discussed in the manuscript (eg, employment/affiliation, grants or funding, consultancies, honoraria, stock ownership or options, expert testimony, royalties, or patents filed, received, or pending), are the following: None.

  ***Funding/Support and role of the sponsor:*** 10.13039/501100002794Swedish Cancer Society (CAN 2023/2807), The Knut and Alice 10.13039/100031203Wallenberg Foundation, 10.13039/501100005934Malmö General Hospital Cancer Foundation, 10.13039/501100004359Swedish Research Council (2021-00859), Lund Medical Faculty (ALF), Skåne University Hospital Research Funds, 10.13039/100012558Skåne County Council’s Research and Development Foundation (REGSKANE-622351), Gösta Jönsson Research Foundation, the Foundation of Urological Research (Ove and Carin Carlsson bladder cancer donation and Astrid and Roland Bengtsson upper tract urothelial carcinoma donation), Maud and Birger Gustavsson Research Foundation, Hjelm Foundation, USVE (Region Skånes Universitetssjukvårdsenheter) Foundation, and Hillevi Fries Research Foundation are acknowledged for their generous financial support.

## Appendix A Supplementary data

Supplementary Data 1 Diagnostic accuracy of PET-CT and CT calculated per anatomical fraction from the side specific template based regional lymphadenectomcy can be found in the supplementary data.
