# Supplementary material for: Evaluating 18F-FDG PET-CT for Regional Lymph Node Assessment in Advanced Upper Tract Urothelial Carcinoma
Source: Eur Urol Open Sci. 2026 May 5;88:74–80. doi: 10.1016/j.euros.2026.04.013 (PMC13157159; doi:10.1016/j.euros.2026.04.013)
Supplement: Supplementary Data 1 — Diagnostic accuracy of PET-CT and CT calculated per anatomical fraction from the side specific template based regional lymphadenectomcy can be found in the supplementary data. [file mmc1.docx]

Supplementary Table 1. Diagnostic accuracy analysis per each removed fraction in the regional lymphadenectomy specimens with an identifiable lymph node on imaging for all patients (n=115) and the subgroup operated without PC (n=78). PET-CT was subjectively interpreted and CT considered positive when short-axis lymph node diameter ≥ 10 mm. rLAE (regional lymphadenectomy); PC (preoperative chemotherapy); CI (95% confidence interval); PPV (positive predictive value); NPV (negative predictive value); LR+ (positive likelihood ratio); LR- (negative likelihood ratio).

|  | PET-CT | | CT | |  |
| --- | --- | --- | --- | --- | --- |
|  | All fractions | Fractions of rLAE without PC | All fractions | Fractions of rLAE without PC |  |
| True positive | 21 | 14 | 9 | 5 |  |
| False negative | 4 | 3 | 16 | 12 |  |
| True negative | 65 | 55 | 78 | 48 |  |
| False positive | 25 | 6 | 12 | 2 |  |
| Diagnostic accuracy (CI) | | | | |  |
| Sensitivity | 84 (64-96) | 82 (57-96) | 36 (18-58) | 29 (10-56) |  |
| Specificity | 72 (62-81) | 90 (80-96) | 87 (78-93) | 97 (89-100) |  |
| PPV | 46 (31-61) | 70 (46-88) | 43 (22-66) | 71 (29-96) |  |
| NPV | 94 (86-98) | 95 (86-99) | 83 (74-90) | 83 (72-91) |  |
| LR+ | 3.0 (2.1-4.4) | 8.4 (3.8-18.5) | 2.7 (1.3-5.7) | 9 (1.9-42.2) |  |
| LR- | 0.2 (0.1-0.6) | 0.2 (0.1-0.6) | 0.7 (0.5-1.0) | 0.7 (0.5-1.0) |  |
